# Multiple time-scale beats in aurora: precise orchestration via magnetospheric chorus waves

**DOI:** 10.1038/s41598-020-59642-8

**Published:** 2020-02-25

**Authors:** K. Hosokawa, Y. Miyoshi, M. Ozaki, S.-I. Oyama, Y. Ogawa, S. Kurita, Y. Kasahara, Y. Kasaba, S. Yagitani, S. Matsuda, F. Tsuchiya, A. Kumamoto, R. Kataoka, K. Shiokawa, T. Raita, E. Turunen, T. Takashima, I. Shinohara, R. Fujii

**Affiliations:** 1 Graduate School of Informatics and Engineering, University of Electro-Communications, Chofu, Tokyo Japan; 2 Center for Space Science and Radio Engineering, University of Electro-Communications, Chofu, Tokyo Japan; 3 Institute for Space-Earth Environmental Research, Nagoya University, Nagoya, Aichi Japan; 4 Graduate School of Natural Science and Technology, Kanazawa University, Kanazawa, Ishikawa Japan; 5 National Institute of Polar Research, Tachikawa, Tokyo Japan; 6 Ionospheric Physics Research Unit, University of Oulu, Oulu, Finland; 7 The Graduate University for Advanced Studies, Hayama, Kanagawa Japan; 8 Department of Geophysics, Graduate School of Science, Tohoku University, Sendai, Miyagi Japan; 9 Institute of Space and Astronautical Science, Japan Aerospace Exploration Agency, Sagamihara, Kanagawa Japan; 10 Sodankylä Geophysical Observatory, University of Oulu, Sodankylä, Finland; 11 Research Organization of Information and Systems, Tokyo, Japan

**Keywords:** Aurora, Magnetospheric physics

## Abstract

The brightness of aurorae in Earth’s polar region often beats with periods ranging from sub-second to a few tens of a second. Past observations showed that the beat of the aurora is composed of a superposition of two independent periodicities that co-exist hierarchically. However, the origin of such multiple time-scale beats in aurora remains poorly understood due to a lack of measurements with sufficiently high temporal resolution. By coordinating experiments using ultrafast auroral imagers deployed in the Arctic with the newly-launched magnetospheric satellite Arase, we succeeded in identifying an excellent agreement between the beats in aurorae and intensity modulations of natural electromagnetic waves in space called “chorus”. In particular, sub-second scintillations of aurorae are precisely controlled by fine-scale chirping rhythms in chorus. The observation of this striking correlation demonstrates that resonant interaction between energetic electrons and chorus waves in magnetospheres orchestrates the complex behavior of aurora on Earth and other magnetized planets.

## Introduction

Humans have always been fascinated by the aurorae, which are natural phenomena that appear in Earth’s upper atmosphere at an altitude of approximately 100 km. They are characterized by luminous photon emissions from atoms and molecules, such as atomic oxygen and molecular nitrogen, excited by energetic charged particles that precipitate from Earth’s magnetosphere^1,2^. The behavior of aurorae represents the prosperity and decline of energetic particles in the magnetosphere due to influences from the Sun’s energy transferred by solar wind. In addition, precipitating electrons and corresponding electric currents, responsible for aurora, are agents that ionize and heat the atmosphere below 100 km in altitude, whose precipitation may lead to destruction of ozone through compositional changes in the mesosphere and stratosphere^3^. Aurorae are commonly observed in the atmospheres of other magnetized planets in the solar system, e.g., the atmospheres of Jupiter and Saturn as imaged by the Hubble Space Telescope^4^. Recent observations from NASA’s Juno mission to Jupiter further provided high-quality multispectral images of Jovian aurorae that have led to a detailed understanding of the atmospheres of magnetized planets energized by charged particles stored in planetary magnetospheres^5,6^.

**Figure 1:**
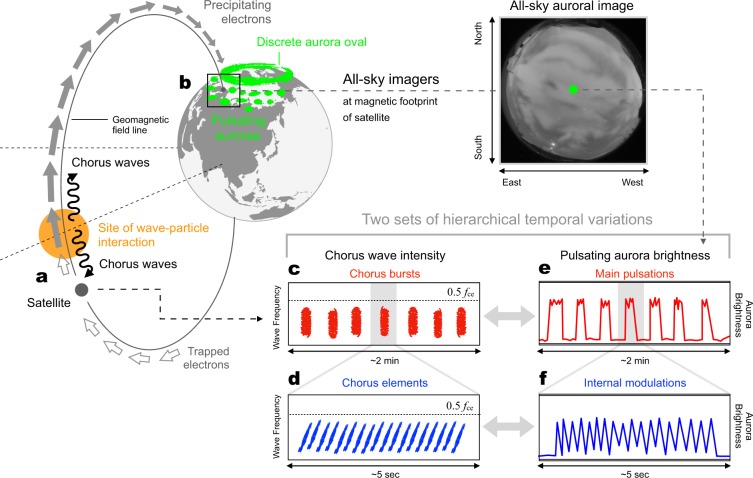
The relationship between magnetospheric chorus waves and a PsA. (**a**) Trapped magnetospheric electrons moving northward (gray open arrows) are scattered via wave-particle interactions with chorus waves (black wavy arrows) at the site of wave-particle interaction near the magnetic equator (orange circle). (**b**) This wave-particle interaction precipitates trapped electrons into the atmosphere in the northern hemisphere (gray filled arrow) along the geomagnetic field and produces patches of PsA. (**c**) The temporal variations in chorus intensity are often characterized by periodic enhancements, which are called chorus bursts. (**d**) A zoomed-in view of a single chorus burst in which several discrete chorus elements are observed. (**e**) Brightness variations in the PsA. A quasi-periodic modulation of optical intensity, with a repetition period ranging from a few seconds to a few tens of seconds, which is known as the main pulsation. (**f**) A zoomed-in view of a single pulse of the main pulsation in which the faster internal modulation luminosity fluctuations are observed.

Past observations have demonstrated that aurorae have a wide variety of dynamic characteristics. One of such features is the so-called “auroral breakup”, which is a sudden and transient expansion of discrete aurora that fills the entire sky for a few tens of minutes^1^. This dynamic behavior is a result of an explosive release of energy that has accumulated in the magnetosphere^2^. Soon after auroral breakups, diffuse and patch-like aurorae appear over a broad region that extends from midnight to dawn and can sometimes last for several hours or longer^1,7^. The majority of such diffuse aurorae are known to beat in a quasi-periodic manner and are commonly referred to as pulsating aurorae (PsAs)^8–10^. Beats in the brightness of PsAs are characterized by a mixture of two distinct periodicities that co-exist hierarchically. One is the “main pulsation”, which is the primary periodicity component ranging from a few to a few tens of seconds. The other is the so-called “internal modulation”, which are much quicker luminosity scintillations (~3 Hz) embedded in a single pulse of the main pulsation^11–13^. Figure 1e,f schematically illustrate how the two independent beats co-exist hierarchically during variations in PsA brightness. That is, ~3 Hz quicker modulations (shown by the blue line) are embedded in a single pulse of the main pulsation (red line) like a Matryoshka doll.

The origin of multiple time-scale beats in PsAs is possibly due to the intermittent occurrence of cyclotron resonance between magnetospheric electrons and natural electromagnetic waves, called “chorus”^14–19^. Chorus waves are primarily generated by free energies carried by electrons injected or convected from the nightside tail of the magnetosphere and drifting closer to the Earth. The energy carried by these electrons is originally transferred from the solar wind into the magnetosphere, stored in the magnetotail, and abruptly released at the time of auroral breakup^2^. Once the chorus waves are generated near the equatorial plane of the magnetosphere, they propagate toward higher latitudes in both hemispheres (as indicated by the black wavy arrows in Fig. 1a). Chorus waves subsequently interact with counter-streaming electrons trapped by the geomagnetic field (Fig. 1a). Through their interactions with chorus waves, a certain fraction of electrons gains sufficient velocity along the magnetic field lines^19^, which enables them to penetrate deeper into the atmosphere^20^ and produce a PsA (Fig. 1b). Theoretical studies have shown that the interactions between magnetospheric electrons and lower-band chorus (LBC) waves, whose frequencies normally lower than 0.5*f*_*ce*_ (where *f*_*ce*_ is the electron gyrofrequency at the source region), provide a viable explanation for the energies typically associated with precipitating PsA electrons, which mostly exceed ~10 keV^14,21^.

Recent studies have attempted to explain the origin of the two beats in PsA by considering the nature of LBC waves^15,22^. Similar to variations in PsA brightness (Fig. 1e,f), LBC wave amplitude modulations are characterized by the superposition of two distinct periodicities: “chorus bursts”, which are trains of quasi-periodic modulations of the wave intensity (Fig. 1c), and much faster modulations, known as “discrete chorus elements”, embedded in single chorus bursts (Fig. 1d)^23^. Previous studies have reported a correlation between the main pulsations of a PsA and chorus bursts (Fig. 1c versus 1e) based on recent simultaneous observations recorded with satellites and ground-based all-sky imagers (ASIs) that have a 3 s temporal resolution^16–19^. However, previous studies did not provide the temporal resolution necessary to probe variations of PsAs with different time scales including the sub-second level internal modulations, which has made it impossible to show conclusive evidence that there is a link between chorus elements and internal modulation (Fig. 1d versus 1f).

To overcome this resolution problem, we developed an ASI system equipped with a highly sensitive cooled Electron-Multiplying Charge-Coupled Device (EMCCD) sensor, which can continuously record 100 images per second with accurate GPS-based timestamps^24^. This sampling rate is 300 times faster than those of conventional observations in previous studies^16–19^. Since 2016, the ASIs have been operative in the Arctic at 3 stations in Scandinavia, 2 stations in Alaska and 1 station in Canada to conduct simultaneous experiments with the wave instruments onboard the newly-launched Japanese magnetospheric satellite Arase^25^. The wave instruments are capable of burst mode operation, which stores raw waveforms of electro-magnetic waves at 64 kHz sampling in limited time intervals. To take full advantage of the limited opportunities to conduct simultaneous experiments, we performed the burst-mode wave experiments when the satellite was situated in the magnetospheric counterpart of the ASI field of view. Such a well-organized “PsA experiment”, by combining the ultrafast EMCCD ASIs with the Arase satellite, enables us to probe the entire spectrum of beating aurora and determine the aurora-chorus correlation not only in the typical second range but also in the sub-second range.

## Results

### Two intervals in the pulsating aurora experiment

**Figure 2:**
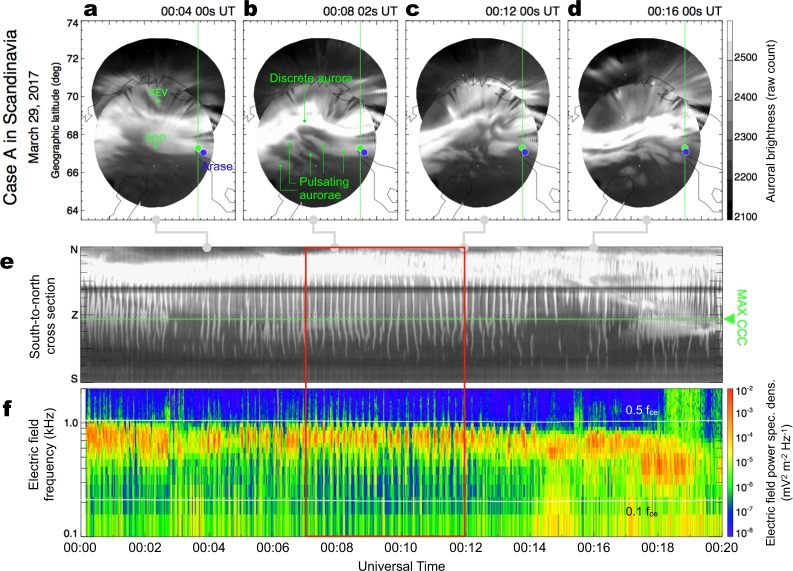
Intense PsA recorded in Scandinavia on March 29, 2017 (Case A). (**a–d**) Successive auroral images from Sodankylä (SOD) and Kevo (KEV), Finland, sampled every ~4 minutes from 00:04:00 to 00:16:00 UT. The original images were mapped to the geographic coordinates assuming an emission height of 110 km. The blue dot in each panel represents Arase’s magnetic footprint estimated using an empirical magnetic field model of the Earth’s magnetic field^26^. The green dot indicates the point at which we obtained the highest cross-correlation coefficient between the chorus intensity from Arase and the auroral luminosity (the detailed procedures to derive this point are provided in the Material and Methods section of the Supplementary Material). (**e**) A time-series of optical intensity along the south-to-north cross-section, including the maximum cross-correlation point (MAX CCC), denoted by the horizontal green line. The cross-section is shown in the upper four panels by the vertical green line. (**f**) A frequency-time diagram of the electric field’s power spectral density from the Arase satellite showing lower-band chorus wave activity. The two horizontal white lines represent 0.5*f*_*ce*_ and 0.1*f*_*ce*_ at the magnetic equator.

In the early stage of the coordinated experiments in March 2017, two intervals of intense PsAs were detected near Arase’s magnetic footprint, including one in northern Scandinavia (Case A) and one in Alaska (Case B). Figure 2 plots a 20-minute-long set of optical and wave observations obtained for Case A on March 29, 2017. In Fig. 2a–d, auroral images obtained from two ASI stations in Finland (Kevo and Sodankylä) illustrate the appearance of a PsA as a cluster of diffuse and relatively dim patches on the southern side of a bright discrete aurora (Fig. 2b). The horizontal scale of such diffuse patches ranges from a few tens to a few hundreds of kilometers. Arase’s footprint (represented by the blue dots in Fig. 2a–d) along the magnetic field line, which was determined using an empirical magnetic field model^26^, remained within the ASI fields of view throughout the interval. Due to accuracy limitations associated with the magnetic field model, Arase’s actual footprint may deviate by a few hundred kilometers from that estimated by the model depending on the magnetosphere’s instantaneous configuration^27^. Alternatively, a point was identified at which the cross-correlation coefficient between the chorus intensity and auroral luminosity time-series was maximized (represented by green dots in Fig. 2a–d). Although the locations of these two footprints were fairly close (the offset was less than 20 km), we, hereafter, regard the maximum cross-correlation coefficient point (“MAX CCC”) as the actual footprint of the satellite following approaches used in previous studies^16–19^.

The temporal variations in auroral brightness sampled along the south-to-north cross-section revealed numerous vertical stripes, which are signatures of the main pulsation (Fig. 2e). The electric field power spectrum (Fig. 2f), which was obtained by the Plasma Wave Experiment/Onboard Frequency Analyzer (PWE/OFA)^28,29^ onboard Arase, revealed prominent wave activity in the LBC frequency range slightly below half the gyrofrequency at the magnetic equator. Specifically, we clearly identified periodic increases in chorus wave intensity in the middle of the time interval (23 enhancements in a 5 min interval from 00:07 to 00:12 UT as highlighted by the red box in Fig. 2f); these increases in intensity are the signatures of chorus bursts. We observed synchronization between the periodic chorus bursts and the series of auroral pulses at the satellite’s footprint (indicated by the green horizontal line in Fig. 2e). Such remarkable agreement over the entire interval supports the previously suggested link between these two periodic phenomena^16–19^.

**Figure 3:**
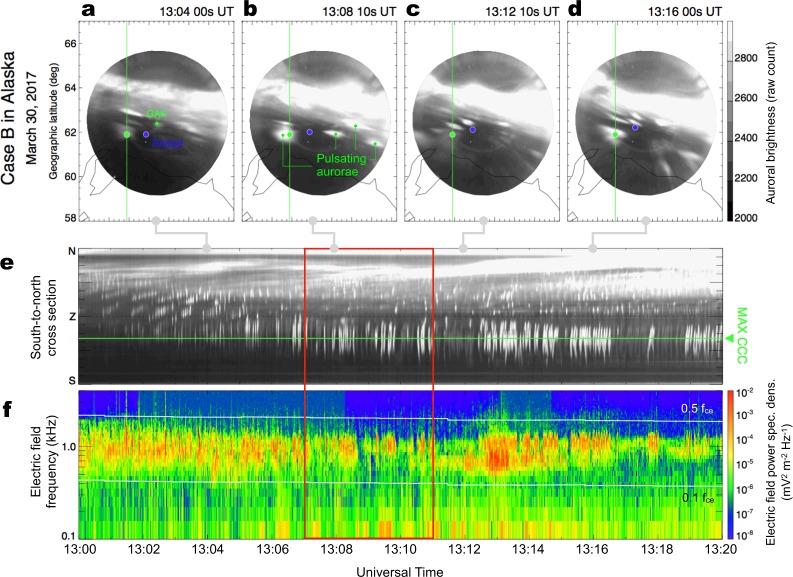
Intense PsA recorded in Alaska on March 30, 2017 (Case B). (**a–d**) Successive auroral images from Gakona (GAK), Alaska, sampled every ~4 minutes from 13:04:00 to 13:16:00 UT. The original images were mapped to the geographic coordinates assuming an emission height of 110 km. The blue dot in each panel represents Arase’s magnetic footprint estimated using an empirical model of the Earth’s magnetic field^26^. The green dot indicates the point at which we obtained the highest cross-correlation coefficient between the chorus intensity from Arase and the auroral luminosity (the detailed procedures to derive this point are provided in the Material and Methods section of the Supplementary Material). (**e**) A time-series of optical intensity along the south-to-north cross-section, including the maximum cross-correlation point (MAX CCC), denoted by the horizontal green line. The cross-section is shown in the upper four panels by the vertical green line. (**f**) A frequency-time diagram of the electric field’s power spectral density from the Arase satellite showing lower-band chorus wave activity. The two horizontal white lines represent 0.5*f*_*ce*_ and 0.1*f*_*ce*_ at the magnetic equator.

A similar enduring correlation for 20 min between PsA and LBC activity was captured again one day later in Case B (Fig. 3). In Fig. 3a–d, we observe small and diffuse auroral patches in the equatorward half of the ASI field of view in Gakona, Alaska. These patches appeared again in the equatorward side of a bright east–west elongating discrete aurora at 63–64 MLAT. Compared with Case A, the spatial scale of the PsA patches is relatively smaller, ranging from 10 to 50 km. Figure 3e displays the temporal variation of the ASI data along the south-to-north cross-section including the MAX CCC point. In the southern part of the field of view, a number of vertical stripes were detected, which are indications of the main PsA pulsation. Contrary to Case A, the pulsating signatures appeared more intermittently, i.e., the appearance of stripes was characterized by a longer periodicity of approximately 1 minute. In addition, the periodicity of the main pulsation looks slightly shorter than that in Case A. During Case A, we observed 23 stripes in ~5 min in the central part of the 20 min interval; thus, the average period of the main pulsation was ~13 sec. In Case B, we observed 26 enhancements during a 3 min interval from 1312 to 1315 UT, which corresponds to a period of ~7 sec. The electric field power spectrum (Fig. 3f) exhibits corresponding periodic bursts of chorus intensity that nearly collocate with the main PsA pulsation.

Before zooming into the detailed correlation between the temporal variations of chorus wave and PsA, we calculate the energy of electrons that can resonate with the observed LBC waves (so-called minimum kinetic energy for cyclotron resonance)^30^. During Case A, we estimated the average background electron density to be 0.73 cm^−3^ by tracing the upper hybrid resonance frequency measured by the PWE High-Frequency Analyzer (PWE/HFA)^31^ onboard Arase. Here, we assume that the electron density at the satellite location is equal to the value at the magnetic equator. By using this electron density together with the magnitude of background magnetic field at the magnetic equator (75 nT), which was calculated by the empirical magnetic field model^26^, and the observed frequency range of LBC wave (0.25–0.5 *f*_*ce*_), we have estimated the resonance energy of the observed LBC wave to range from 5.2 to 33 keV, which well explains the typical energy of precipitating electrons causing PsA^20,22^. Similar calculation has been done for Case B by using the average background electron density at the location of satellite (1.52 cm^−3^), the magnitude of background magnetic field at the magnetic equator (145 nT), and the observed frequency range of LBC wave (0.15–0.4 *f*_*ce*_). The derived resonance energy ranges from 18 to 141 keV. This range of energy seems to be slightly higher than the typical energy of PsA electrons^20,22^ while previous studies^32^ showed wide energy electron precipitations from tens keV to sometimes more than 100 keV associated with PsA. These estimations confirm that the LBC waves observed by the satellite were actually responsible for the scattering of electrons that caused the PsA emission seen from the ground during the two case examples.

### Close-up view of the “main pulsation”

**Figure 4:**
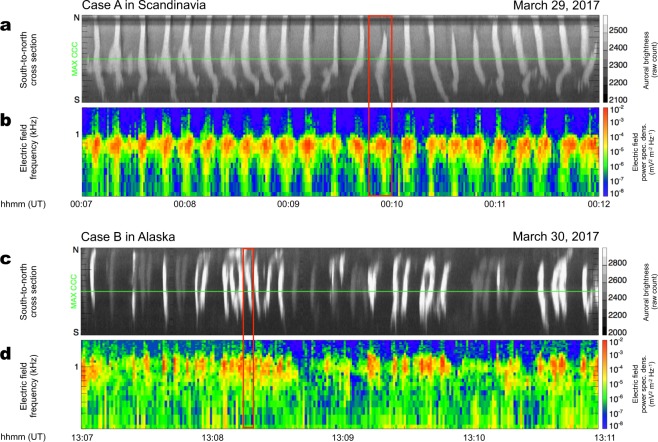
A direct comparison between the main PsA pulsations and chorus bursts. (**a,b**) A zoomed-in view of the optical and wave data for a 5 min interval from Case A, which is highlighted by the red box in Fig. 2e,f. (**c,d**) A zoomed-in view of the optical and wave data for a 4 min interval from Case B, which is highlighted by the red box in Fig. 3e,f.

The correlation between the main pulsation and chorus bursts is more clearly depicted in Fig. 4, which focuses on a central time interval for each of the two cases, respectively (highlighted by the red box in Figs. 2e,f and 3e,f). For Case A (Fig. 4a,b), we observe a clear visual correspondence and find that a one-to-one mapping always occurs between each PsA pulse and chorus burst. Supplementary Movie S1 more clearly depicts this correlation, where the luminosity of the auroral patches intensifies during the chorus bursts. At the same time, the movie demonstrates that most of PsA patches during Case A moved during single pulses of main pulsation. PsA showing such horizontal motion has been recognized as streaming or propagation type PsA in the past literature^33,34^. A similarly strong correlation was observed in Case B (Fig. 4c,d), which adds further weight to the notion that chorus bursts directly regulate the main PsA pulsations. The one-to-one mappings between chorus bursts and the main PsA pulses allow us to evaluate the correlation between discrete chorus elements and internal modulations by zooming in on the ultrafast optical and high-time-resolution waveform data at 64 kHz sampling from the Plasma Wave Experiment/Wave Form Capture (PWE/WFC)^28,29^ onboard Arase during the two limited time periods highlighted by the red boxes in Fig. 4.

### Further zoomed-in view of the “internal modulation”

**Figure 5:**
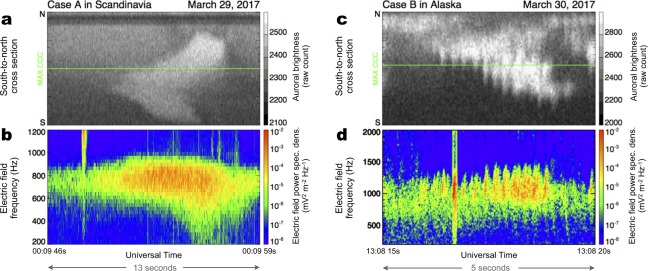
A direct comparison between the internal PsA modulations and chorus elements. (**a,b**) A zoomed-in view of (**a**) the single chorus burst structure and (**b**) the corresponding PsA pulse from Case A. This picked up interval is highlighted by the red box in Fig. 4a,b. (**c,d**) A zoomed-in view of (**c**) the single chorus burst structure and (**d**) the corresponding PsA pulse from Case B. This interval is highlighted by the red box in Fig. 4c,d.

Using the 64 kHz sampling burst mode wave data, which was collected by the PWE/WFC onboard Arase, the fine-scale characteristics of the phenomena were observed by isolating a single burst of the chorus and corresponding PsA pulse. Figure 5 shows close-up views of a single chorus burst and the corresponding PsA pulse for two short intervals highlighted by the red boxes in Fig. 4. For Case A, there is no apparent signature of discrete chorus elements (Fig. 5b). Such less-structured chorus emissions are known as “hiss-like” emissions^35^ and are thought to result from densely populated chorus elements due to strong and continuous non-linear growth of whistler-mode waves^36^. In accordance with such a less-structured chorus emission, typical internal modulation signatures, with an ~3 Hz periodicity, were not observed in the optical data (Fig. 5a). For Case A, the estimated magnetic footprint of the satellite was located close to the eastern edge of the field of view of the ASI (Fig. 2a–d). If the optical emission of the PsA patches around the footprint has a certain thickness in altitude, one may think that internal modulation is smeared out by integrating the optical pulsations in different locations obliquely. In this case, however, it should be emphasized that the corresponding chorus emission is also less structured, which suggests that the internal modulation should have really been absent and such a blurring effect is not probable. Unlike that in Case A, the chorus burst in Case B (Fig. 5d) reveals a fine-scale structure comprised of several internal rising-tone elements^23^. Given the close correspondence with the well-structured emission depicted in Fig. 5d, the optical data collected from the satellite’s footprint show successive brightness modulations with a periodicity of 3–4 Hz (Fig. 5c), which are indicative of internal PsA modulation^11–13,22^. Note that a short-lived and broadband feature in the first half of the dynamic spectra in Fig. 5d is an artificial instrumental noise. Similar short-lived wave signature is seen for Case A (Fig. 5b), which is not considered as an instrumental noise. Since we do not see any corresponding signature in the ground-based optical data, the origin of this broadband wave feature is still under investigation.

**Figure 6:**
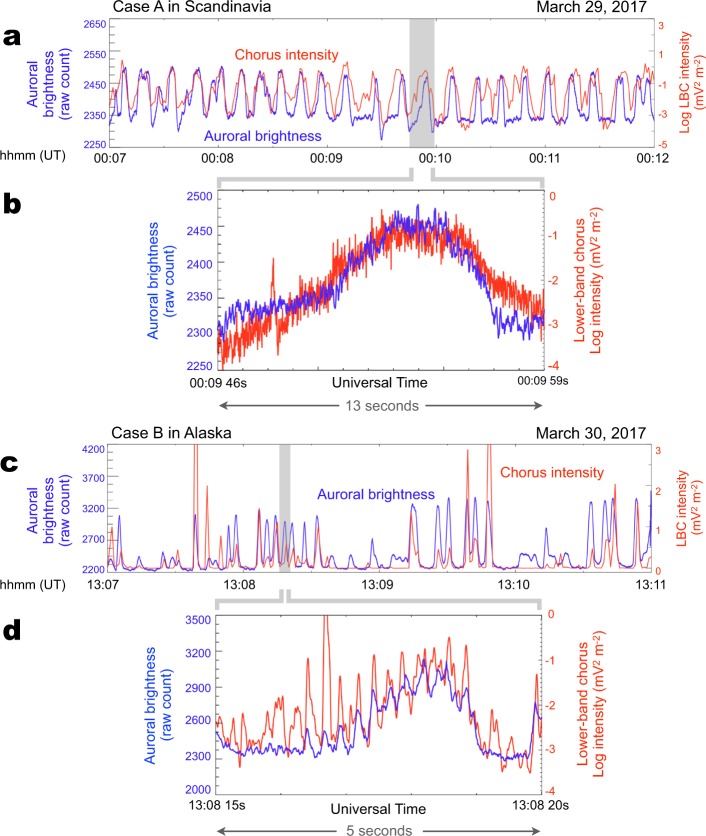
A direct comparison between the time-series of PsA luminosity and chorus intensity. (**a,c**) The blue line represents the auroral luminosity time-series at the MAX CCC point for (**a**) Case A and (**c**) Case B. The red line denotes the integrated wave power in the LBC wave frequency range (400 to 1200 Hz). (**b,d**) A zoomed-in view of the optical and wave data for (**b**) Case A and (**d**) Case B. The blue line represents the auroral luminosity time-series at the MAX CCC point while the red line denotes the integrated wave power over the LBC frequency range. The temporal variations in these two signatures are coherent (at a 0.28 s delay) for Case B.

To compare the PsA and LBC temporal variations in a more direct manner, we plotted both datasets as time-series in Fig. 6. For Case A, all the main PsA pulses have corresponding bursts in the chorus data (Fig. 6a). Figure 6b is a zoomed-in view of one of the main pulses observed slightly before 00:10 UT. Both time-series are characterized by fast fluctuations, which is a sign of densely populated chorus elements, as well as the resulting slight modulations within a single PsA pulse. However, identification of the typical sub-second variations in both datasets is rather difficult. For Case B, there was again a good correspondence between the main pulsation and chorus burst (Fig. 6c). A direct comparison between auroral brightness and chorus intensity in a 5 s interval shows an excellent one-to-one correlation between chorus elements and internal modulation (Fig. 6d). To obtain the best correlation between the two time-series, we must consider a 0.28 s delay for the LBC data, which is discussed in the following section.

The remarkable correlation between the internal modulation and chorus elements for Case B (Fig. 6d) is more clearly observed in Supplementary Movie S2, in which the chorus data are incorporated as sounds by converting the raw waveform from PWE/WFC to sound. The exact one-to-one correspondence between the rhythmic beating in auroral brightness and the periodic chirping of chorus elements supports the hypothesis that each chorus element directly controls the sub-second scintillation of PsA. One of the primary reasons as to why we were able to capture striking correlation between the two sub-second variations is the use of high-resolution ASIs (with 100-Hz sampling, which is 300 times faster than the sampling frequencies used in previous studies)^24^ deployed at the footprints of the satellite and the waveform data by PWE/WFC onboard the Arase satellite. This capability also demonstrated that less-structured chorus emissions produce a PsA without internal modulation (Case A; Figs. 5a–b and 6b).

## Discussion

We have demonstrated that there is a direct association between the multiscale temporal variations in chorus wave power and aurorae luminosity. We identified, simultaneously, two sets of one-to-one correlations in the PsA generation process. The first correlation exists between chorus bursts and the main pulsation (Fig. 1c versus 1e). The second exists between discrete chorus elements embedded in a burst and internal PsA modulation (Fig. 1d versus 1f). These correlations confirm the hypothesis that the two distinct PsA periodicities are fully orchestrated via similar hierarchical temporal variations in magnetospheric chorus waves. When the two time-series are compared in Fig. 6d, we use a 0.28 s time delay for chorus intensity. This delay time was derived by performing cross-correlation analysis between auroral brightness and chorus intensity. At the time of this correlation analysis between internal modulations and chorus elements, we first identified a pixel of maximum correlation between main pulsation and chorus burst (i.e., MAX CCC in Fig. 3). After specifying the pixel, we again applied the cross-correlation analysis to the time-series of internal modulation at the point of MAX CCC and burst mode chorus data with various time lags. The delay time of 0.28 s is consistent with the travel time of PsA electrons, having the minimum resonance energy (~18 keV) estimated in the previous section, from the magnetosphere’s equatorial plane to PsA altitude at ~100 km along the field line^22^, which further supports the causality between discrete chorus elements and sub-second PsA variation.

Previous ground-based observations indicated that approximately 50% of PsAs display internal modulation^11^. Thus far, however, the process that controls the existence or absence of such sub-second level fast modulations has been unclear. In the last decade, the relationship between discrete chorus elements and internal modulation of PsA was theoretically predicted^22^, and it was also inferred from ground-based simultaneous observations^37^. More recently, such a correlative behavior was captured for the first time during a similar event of ground-satellite conjunction^38^. By comparing the two cases showing clear and unclear internal modulations of PsA, we succeeded in proving that less-structured “hiss-like” chorus emission^35,36^ produces PsAs without the ~3 Hz internal modulation. Although we did not observe the well-known ~3 Hz modulation, the burst mode wave data in Fig. 6b indicate that tiny internal structures in the hiss-like emission still occur. However, this fast fluctuation is unable to produce a clear internal PsA modulation signature due to finite dispersion in the travel time of electrons with different energies^22^. The other possible reason for the absence of internal modulation for Case A is a blurring effect due to the characteristic motion of PsA patches. As stated above, most of PsA patches during Case A moved during single pulses of main pulsation. We cannot identify the motion of the chorus structure (i.e., the source region of wave-particle interaction) in the magnetosphere because the satellite observation is basically a single point measurement. However, if the chorus source region moves near the magnetic equator, chorus elements observed by the satellite in the off-equatorial region and corresponding internal modulation could be smeared out by the superposition of temporal and spatial variations.

The comparisons between the two cases imply a direct link between the morphology of PsA seen from the ground and the characteristics of chorus in the magnetosphere. This means that diversity into the morphological characteristics of PsAs is introduced directly by the nature of chorus waves in the magnetosphere. Periodic chorus wave variations that occur over multiple timescales are universal for not only Earth’s magnetosphere but also for the magnetospheres of Jupiter, Saturn^39^, and their moons^40^. Diffuse aurorae are also commonly observed in the atmospheres of these planets, e.g., based on observations from the recent Juno mission to Jupiter^6^. However, due to imaging observations that have an insufficient temporal resolution, we are unable, at present, to understand the temporal variations in these diffuse emissions. The results of this study imply that the chirping sounds produced by plasma waves in a planetary magnetosphere are direct indications of beating diffuse aurorae on that planet. This knowledge will enable us to investigate temporal variations in diffuse planetary aurorae using plasma wave observations during future missions.

## Methods

### High time-resolution all-sky imagers

To perform this coordinated satellite and ground comparison study, we deployed four high-speed all-sky imagers (ASIs) to conduct high time-resolution measurements of pulsating aurorae at the possible magnetic footprints of the Arase satellite. The ASI system comprises a fish-eye lens, interference optical filter, and an electron multiplying charge-coupled device (EMCCD) detector, which enabled the collection of 100 all-sky images at a spatial resolution of 256 × 256 without any significant frame loss. These 100-Hz ASI sampling observations enable us to capture internal modulation in pulsating aurora that is comparable with high time-resolution waveform data from the satellite. The ASI had an RG665 interference optical filter, which is sensitive only to prompt auroral emissions from molecular nitrogen bands (so-called N_2_ first positive band extending from 650 to 700 nm wavelength range). This selective filtering allows us to remove contamination from other slow (delayed) emissions, e.g., an emission at 557.7 nm, which is a green line emission from atomic oxygen; thus, we are able to compare satellite wave and optical data without taking into account blurring effects within the temporal variation of a pulsating aurora.

### Estimation of the real footprint via cross-correlation analysis

Due to uncertainty associated with the magnetic field model^26^, locating the satellite’s magnetic footprint using only the model is difficult. In previous studies that compared satellite/ground data for pulsating aurorae^16–19^, the actual footprint was estimated using a cross-correlation analysis of the chorus intensity and pulsating aurora luminosity time-series. Following this approach, we estimated Arase’s footprint during the two conjunction intervals. Two examples of the footprint estimation process are shown in Supplementary Fig. S1 (Case A) and S2 (Case B) online. For Case A, the actual estimated footprint (Point of Maximum Correlation = PMC in Fig. S1d) was nearly identical to that derived from the magnetic field model (denoted by ARASE in Fig. S1c,d). In contrast, for Case B, the maximum cross-correlation point (PMC in Fig. S2d) is located in the western region of the model footprint, which implies that the magnetic field model was incorrect for this case. During the detailed comparison process used in this study, we selected a point in the ASI field-of-view (FOV) where the cross-correlation coefficient between the chorus intensity and optical luminosity acts as the actual satellite footprint.

### Chorus burst observation

The Arase satellite PWE (Plasma Wave Experiment)^28^ instrument routinely observes electric and magnetic fields at 64 kHz sampling. However, downlinking all raw waveform data is impossible. The Plasma Wave Experiment/Onboard Fourier Analyzer (PWE/OFA)^29^ stores and downlinks the wave data as spectra with a nominal temporal resolution of 1 s, which is sufficient to identify temporal variation in chorus bursts. To observe the detailed internal structure of a chorus burst, the Plasma Wave Experiment/Wave Form Capture (PWE/WFC)^29^ records raw waveforms at 64 kHz sampling in limited periods during satellite/ground conjunctions. Based on these dedicated chorus burst observations, we are able to observe the rapid chorus element variations that are embedded in a single chorus burst. Raw waveforms captured by PWE/WFC were recorded in Movie S2 as audio data.

## Supplementary information

Supplementary Information.

Supplementary Movie S1.

Supplementary Movie S2.

## Data Availability

The EMCCD ASI images and chorus wave data from Arase used in this study are available from the ERG Science Center operated by ISAS/JAXA and ISEE/Nagoya University (https://ergsc.isee.nagoya-u.ac.jp/data_info/index.shtml.en). The present study analyzed the PWE/OFA L2 v.02.01 data.
