# Supplementary material for: Multiple time-scale beats in aurora: precise orchestration via magnetospheric chorus waves
Source: Sci Rep. 2020 Feb 25;10:3380. doi: 10.1038/s41598-020-59642-8 (PMC7042315; doi:10.1038/s41598-020-59642-8)
Supplement: Supplementary file 1 — Supplementary Information. [file 41598_2020_59642_MOESM1_ESM.pdf]

SUPPLEMENTARY INFORMATION

Multiple time-scale beats in aurora: precise orchestration via magnetospheric chorus waves

Keisuke Hosokawa, Yoshizumi Miyoshi, Mitsunori Ozaki, Shin-Ichiro Oyama,
Yasunobu Ogawa, Satoshi Kurita, Yoshiya Kasahara, Yasumasa Kasaba, Satoshi Yagitani,
Shoya Matsuda, Fuminori Tsuchiya, Atsushi Kumamoto, Ryuho Kataoka, Kazuo Shiokawa,
Tero Raita, Esa Turunen, Takeshi Takashima, Iku Shinohara, Ryoichi Fujii

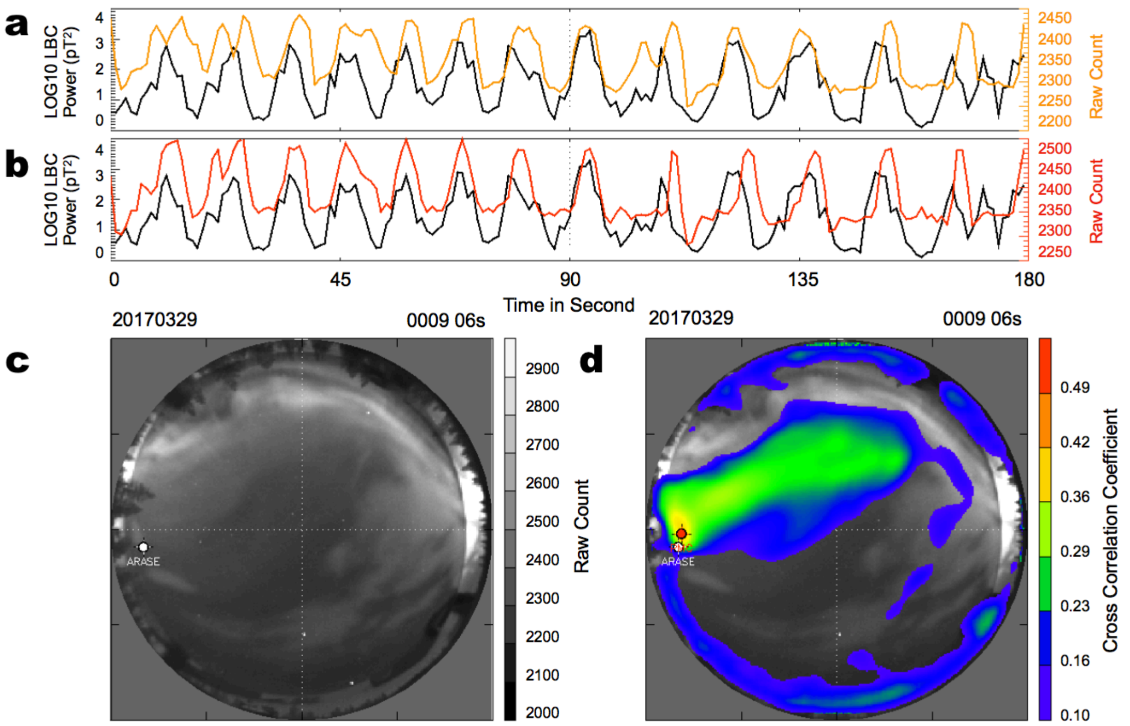

Fig. S1. A cross-correlation analysis for Case A in Scandinavia on March 29, 2017. **a** The chorus power (black line) and optical intensity time-series from the ASI in Sodankylä at the modelled magnetic footprint of Arase (orange line) during a 3 min interval from 00:07:36 to 00:10:36 UT. **b** The chorus power in the magnetic field (black line) and optical intensity time-series at the MAX CCC between the two time-series (red line). **c** An ASI image taken in the middle of the 3 min interval shown in **a-b**. **d** The coefficient distribution obtained in the cross-correlation analysis superimposed on the ASI image shown in **c**.

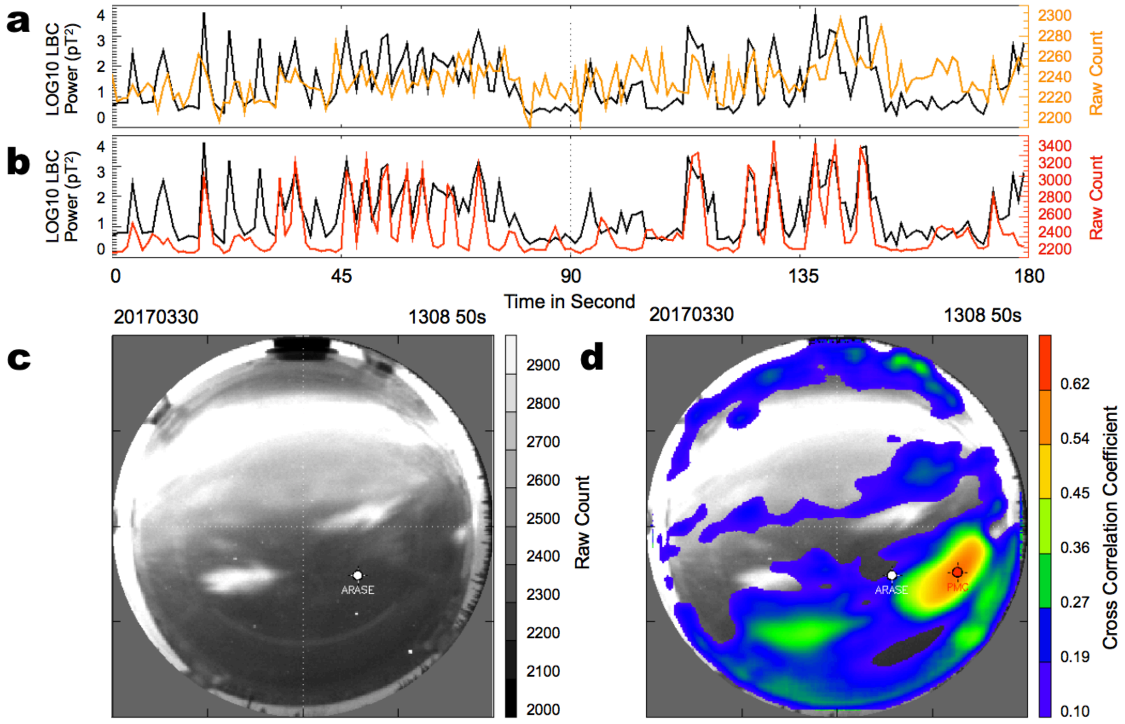

Fig. S2. A cross-correlation analysis for Case B in Alaska on March 30, 2017.

a The chorus power (black line) and optical intensity time-series from the ASI in Gakona at the modelled magnetic footprint of Arase (orange line) during a 3 min interval from 13:07:20 to 13:10:20 UT. **b** The chorus power in the magnetic field (black line) and optical intensity time-series at the MAX CCC between the two time-series (red line). **c** An ASI image taken in the middle of the 3 min interval shown in **a-b**. **d** The coefficient distribution obtained in the cross-correlation analysis superimposed on the ASI image shown in **c**.

Movie S1.

A movie showing the one-to-one correlation between the main pulsation of PsA and chorus bursts. The top left panel shows the optical data from Sodankylä, Finland during a 3.5 min interval from 00:06:45 UT to 00:10:15 UT on March 29, 2017. The top right panel is a zoomed-in image taken near the satellite footprint. The middle panel presents the temporal variation in optical data along the green line, which is the south to north cross-section from the ASI in the top two panels, where the main pulsation is seen as a series of vertical stripes. The bottom panel is the frequency-time diagram of chorus data from Arase, in which the chorus bursts are seen as periodic enhancements of wave intensity. The moving bar traces the progression of time in the video.

Movie S2.

A movie showing the one-to-one correlation between the internal modulation of PsA and discrete chorus elements. The top left panel shows the optical data from Gakona, Alaska during a 5 s interval from 13:08:15 UT to 13:08:20 UT on March 30, 2017. The top right panel is a zoomed-in image near the satellite footprint. The middle panel shows the temporal variation in optical data along the green line in the top two panels, where we detected internal modulation as successive enhancements in luminosity. The bottom panel presents a frequency-time diagram of the chorus data from Arase, which contains captured periodic rising traces of chorus elements. The moving bar traces the progression of time in the video. Chorus data are compiled as audio material, which is raw wave data from the Arase satellite that were converted to sound.
